# Identification of an ancient endogenous retrovirus, predating the divergence of the placental mammals

**DOI:** 10.1098/rstb.2012.0503

**Published:** 2013-09-19

**Authors:** Adam Lee, Alison Nolan, Jason Watson, Michael Tristem

**Affiliations:** 1 Imperial College London, South Kensington Campus, London SW7 2AZ, UK; 2 Imperial College London, Silwood Park Campus, Buckhurst Road, Ascot, Berkshire SL5 7PY, UK

**Keywords:** retrovirus evolution, mammalian evolution, selfish genetic elements, mammalian-wide interspersed repeat, genomics, paleovirology

## Abstract

The evolutionary arms race between mammals and retroviruses has long been recognized as one of the oldest host–parasite interactions. Rapid evolution rates in exogenous retroviruses have often made accurate viral age estimations highly problematic. Endogenous retroviruses (ERVs), however, integrate into the germline of their hosts, and are subjected to their evolutionary rates. This study describes, for the first time, a retroviral orthologue predating the divergence of placental mammals, giving it a minimum age of 104–110 Myr. Simultaneously, other orthologous selfish genetic elements (SGEs), inserted into the ERV sequence, provide evidence for the oldest individual mammalian-wide interspersed repeat and medium-reiteration frequency interspersed repeat mammalian repeats, with the same minimum age. The combined use of shared SGEs and reconstruction of viral orthologies defines new limits and increases maximum ‘lookback’ times, with subsequent implications for the field of paleovirology.

## 1. Introduction

Endogenous retroviruses (ERVs) are proviral DNA elements that insert into the germline cells of the host organism, being vertically transmitted from one generation to the next [1]. Intact elements usually comprise retroviral structural genes, an encoded polymerase and viral envelope, flanked on either side by long terminal repeats (LTRs). Over time, germline ERV insertions accrue an increasing number of irreversible mutations in their DNA, often leading to inactive retroviral elements, defective in their ability to produce infectious virions [2]. In some cases, ERV insertions remain in the genome as ‘molecular fossils’ of ancient infection events, and can be used to better understand and study the evolution of both retroviruses and their hosts [3]. Upon integration, ERVs likely retain greater genomic conservation than their exogenous counterparts, being subjected only to the host's rate of genome evolution [4].

ERVs are classified into families, each of which contains viruses thought to have derived from a single, initial infection event. Many human endogenous retroviruses (HERVs), for instance, stem from an infection event of an ancestral mammalian genome [5], formerly suggested to have occurred as much as 70 Ma in one particular family [6]. This family is HERV-L (HERV with leucine tRNA primer), discovered in 1995, with related sequences having been found at copy numbers between 1 and 200 in the genomes of many mammals [7]. As a family, ERV-L thus appears to have been active for much of mammalian evolution. Unusually for a retrovirus, ERV-L does not contain an envelope gene.

Viral genomes are typically prone to high rates of nucleotide substitutions, often resulting in many lineages becoming extinct relatively quickly [8]. However, unlike other viruses, retroviruses must integrate into the genome of their host as part of their replication cycle [9]. If this integration event occurs in a germ cell, then the retroviral genome becomes endogenized, and its mutation rate is generally confined to the host organism's neutral rate of evolution (with the exception of rare cases in which the retroviral element becomes co-opted) [10,11]. ERVs can thus provide a long-term record of host and virus evolution.

In order to date these integrated retroviruses, sequence divergence between the two LTRs can be measured [5]. While useful in estimating the age of recent insertions, this method is not as easily applicable to estimating the age of ancient ERVs, since these are often highly mutated. A more efficient procedure to obtain a minimum age of certain ancient ERVs is to identify orthologous elements in related taxa. The presence of orthologous ERVs in two or more host species implies that the initial infection event predates the divergence of those species [12]. Furthermore, co-divergence or co-speciation studies of viruses and their hosts can also indicate ancient associations. This approach, based largely on the study of extant viruses, and not a panel of ancient ERVs, has been used to indicate that some foamy viruses originated more than 100 Ma [13].

While there have been few studies investigating the age of viral insertions using orthologous elements, one study involved dating orthologous retroviruses in the European hare. The retrovirus, termed rabbit endogenous lentivirus type K, was found inserted at the same genomic locus across the order Lagomorpha, giving the element a minimum age of 12 Myr [14]. While orthologies were not used for dating, cophylogenetic analysis of a retroviral element discovered in the genome of a coelacanth gave an estimated age of 407 Myr for foamy-like viruses [15]. Another study involved reconstructing orthologies for several non-retroviral endogenous virus elements, one of which was a virus of the family *Bornaviridae*, found to have a minimum age of 93 Myr; members of the *Parvoviridae* and *Circoviridae* were also examined, with minimum ages of 30 and 60 Myr, respectively [16].

In addition to ERVs, host genomic DNA can also be subjected to the insertion of other selfish genetic elements (SGEs) such as short interspersed elements (SINEs) and long interspersed elements. These SGEs can amplify themselves within the genome, often to the detriment of the host organism, which may lead to their eventual deactivation or deletion via selection. However, if SGEs are neutral to the host, then they can reach fixation in the genome and, much like ERVs, can be passed down vertically across many generations, often making ancient SGE insertions useful in evolution and dating studies [17]. Among the most prevalent SGE families in the Mammalia, are mammalian-wide interspersed repeats (MIRs). Estimated to have started amplifying within mammalian genomes during the Mesozoic era, this family of repeats can be found at copy numbers as high as 10^7^. SGE orthologies have also been reconstructed across several mammalian superorders. Namely, an orthologous MIR insertion was demonstrated occurring at nine loci within humans and non-primate mammalian sequences dating back 65 Myr which, together with the application of a molecular clock, suggested an age of approximately 130 Myr for this family of repeats [18].

Mammalian genomes also contain large numbers of medium-reiteration frequency interspersed repeats (MERs). In particular, the MER3 family exhibits the highest diversity, with poor conservation found between elements [19]. While orthologies have not been reconstructed to date, MER3 elements are thought to be at least 55 Myr old [20]. Another family of SGEs, with a distribution across the placental mammals, is mammalian apparent LTR-retrotransposons. An individual element found in both humans and mice, suggested an age of 80–100 Myr for this family, although, as of yet, no definitive orthology has confirmed this [21]. Likely older still are the *Ty3/Gypsy* family of LTR-retrotransposons, where related sequences were observed in both vertebrates and invertebrates. Phylogenies of these sequences suggested a potential age of 600 Myr for this family [22].

In this investigation, we examined a member of the ERV-L family and, via orthology reconstruction, confirmed that the element probably integrated before the divergence of placental mammals, making this the oldest individual virus described to date. Furthermore, three SGE insertions within the ERV-L were found across all, or nearly all, of the studied taxa, making these the oldest individual MIR and MER3 elements described. We further discuss some of the difficulties in identifying ancient retroviral insertions, while estimating possible maximum ‘lookback’ times for ERVs, and the corresponding implications for the field of paleovirology, in general.

## 2. Material and methods

### (a) Element retrieval and sequence alignment

The published HERV-L reference sequence (X89211; [7]) was retrieved from NCBI's Nucleotide database, and used to perform BLAST searches against the genomes of various mammals. The algorithm selected was BLASTN. Initially, one particular ERV-L sequence was identified in several mammalian superorders; preliminary analysis of LTRs and sequence flanking the viral insertion in these species suggested possible orthology. This particular ERV-L element was therefore chosen as the subject of further characterization.

We used this newly identified human ERV-L element as a probe, *in silico.* A further 10 homologous retroviral sequences were retrieved from different mammalian hosts through various BLAST searches against NCBI's WGS, HTGS and refseq_genomic databases [23]. It should be noted that many of the taxa selected for investigation have low rates of neutral evolution. The viral sequences were extracted, together with sequences flanking the 5′ and 3′ LTRs of the elements, and were run through NCBI's open reading frame (ORF) finder, to screen for any intact retroviral ORFs. The mammals included in this study span superorders I–IV, and include the pig (*Sus scrofa*), horse (*Equus caballus*), Seba's short-tailed bat (*Carollia perspicillata*), large flying fox (*Pteropus vampyrus*), human (*Homo sapiens*), chimpanzee (*Pan troglodytes*), nine-banded armadillo (*Dasypus novemcinctus*), African elephant (*Loxodonta africana*), West Indian manatee (*Trichechus manatus*), aardvark (*Orycteropus afer*) and golden mole (*Chrysochloris asiatica*). In addition to this, BLAST searches of the available marsupial genomes were also conducted.

An initial alignment was constructed from approximately 1.2 kilobase (kb) of 5′ flanking sequence and 70 basepair (bp) of the 5′ LTR from the start of the element. Sequences were then aligned using EBI's multiple sequence comparison by log-expectation (MUSCLE) algorithm [24], with resulting alignments being adjusted and corrected manually. Sequence conservation was also recorded at each individual site, and plotted as a histogram beneath the alignment. This was repeated for approximately 2 kb of 3′ flanking sequence and 70 bp of the associated 3′ LTR. Sequences demonstrating orthology were retained, and the corresponding viral insertion in each species was loaded into a third alignment. The full-length alignments are available in the electronic supplementary material.

The flanking sequence was trimmed, and the complete viral elements, from the 5′ end of the 5′ LTR through to the 3′ end of the 3′ LTR, were individually run through RepeatMasker [25] with the appropriate species-specific repeat settings applied, in order to identify SGEs, including regions of the ERV-L itself. Identified elements and repeats were then annotated onto the sequence using CLC's main workbench. Elements that appeared to be anomalously assigned by RepeatMasker were investigated at the nucleotide level, and manually corrected. Once annotated, sequences were aligned again using MUSCLE, and the alignments were carefully edited and adjusted manually, using NCBI's bl2seq and ClustalW to guide manual alignment of small fragments of the viral element. Following alignment, any insertions in two species or fewer that amounted to 10 bp or more were deleted (deleted regions are specified in the electronic supplementary material).

Subsequent to the annotation of the sequences, an additional LTR structure was identified in the Afrotheria, approximately 2300 bp into the viral alignment. In order to verify that this was a solo-LTR structure, approximately 400 bp of both 5′ and 3′ LTRs paralogous to the published HERV-L sequence were retrieved from the human genome. In combination with the Afrotherian LTR, these were used to construct a maximum-likelihood phylogeny, in order to confirm whether this was in fact a solo-LTR (A. Lee & M. Tristem 2013, unpublished data).

### (b) Sequence identities

In order to numerically evaluate and represent homology between the orthologues, two sets of pairwise comparisons were made between species, to give observed percentage identities across all pairs of taxa. First, a 997 bp region from the start of the element was extracted, and all gaps were removed. This was repeated for a second, 830 bp region starting at position 4417 (see the electronic supplementary material). Shared, pairwise sequence identities were then computed using CLC main workbench.

### (c) Phylogenetic analysis

A 1 kb aligned section of ERV internal region downstream of the 5′ LTR was used as the basis of phylogenetic analysis. The region used is designated region 1. All species were included, apart from Seba's short-tailed bat and the flying fox, both of which had insufficient sequence data at this region. A maximum-likelihood phylogenetic tree was built from this region using the TN93 substitution model, performed in MEGA [26]. Branch support was in the form of 10 000 bootstrap replicates. A host phylogeny was derived from the literature in order to construct a cophylogeny. Minimum divergence dates for the various host taxa were also obtained from the literature.

A second phylogeny was built from a segment of ERV internal region (from positions approx. 3220–3820; designated region 2), in order to confirm observed phylogenetic relationships resolved by the first tree. The same substitution model and bootstrapping were used. This was then plotted onto a cophylogeny, together with the mammalian tree. The Afrotheria were excluded from this phylogeny, as they did not align sufficiently. This second cophylogeny has been made available in the electronic supplementary material.

### (d) Estimating initial integration date

To determine how long before the radiation of placental mammals the initial integration event occurred (assuming Eutherian divergence at 104 Ma), we calculated the percentage of non-identical residues between the LTRs of three combinations of species, representing each of the four mammalian superorders. These included human (superorder III) against elephant (superorder I), human against armadillo (superorder II) and human against horse (superorder IV).

The orthologous 5′ LTRs of the human and elephant ERV-L element were run through the EMBOSS Needle sequence alignment algorithm [27], creating a pairwise alignment. Elephant 5′ and human 3′ LTRs were also subsequently aligned using Needle, and the percentage of non-identical bases was obtained. Subtracting the former value from the latter value gives an approximate estimate for the amount of LTR divergence occurring prior to the initial placental mammal speciation events. This value was then divided by the neutral mutation rate of the mouse genome, which then allows an estimated date of the initial germline integration event to be calculated. These calculations assume that the LTRs are subjected solely to a steady rate of host genome evolution over time. The calculations were then repeated for the remaining species combinations.

### (e) *d*_N_/*d*_S_ ratios

The complete MuERV-L sequence (accession number Y12713) was obtained from NCBI's Nucleotide database, and a BLAST search of this sequence against the investigated ERV-L element was performed, to retrieve the areas with highest homology in the human sequence. These individual matching regions were compiled into a separate alignment, and all leading and trailing MuERV-L sequence between the blocks demonstrating homology were deleted (while maintaining an ORF in the MuERV-L sequence, and therefore the presumed ORFs in the original ERV-L sequences). Species included in the alignment comprised armadillo, human, Seba's short-tailed bat, flying fox, chimpanzee, pig and horse. The regions aligning to MuERV-L fell within the approximately 2.5 kb of poorly aligning sequence in the Afrotheria, so the corresponding four species were omitted.

The alignment was then used to calculate codonwise *d*_N_/*d*_S_ ratios using HyPhy [28] in MEGA [26], together with *p*-values; if significant (less than 0.05), then these could be used to reject a null hypothesis assuming the sequences are evolving under neutrality [28,29]. A total of 108 amino acid sites were deduced from the alignment, and both *p*-values and *d*_N_/*d*_S_ ratios were averaged to give individual values.

### (f) Synteny analysis

Analysis of chromosome-wide syntenic genes surrounding the ERV-L integration was performed using ENSEMBL's SyntenyView, with available species for analysis, including human, pig, horse and chimpanzee. The human ERV-L element was found to be located on chromosome 17; synteny across this chromosome was visualized together with the corresponding chromosomes in the pig, horse and chimpanzee. Other host taxa were not available for analysis in SyntenyView.

## 3. Results

### (a) Flanking sequence similarity between ERV-L elements

We identified an ERV-L sequence in 11 placental mammals by BLAST searches of genomic databases. While the available genomes of several marsupials were also screened, these returned negative results. Most of the examined host species were specifically selected for their relatively low neutral mutation rates, because this would be expected to best preserve sequence similarity between any orthologous viruses and other SGEs.

**Figure 1.**
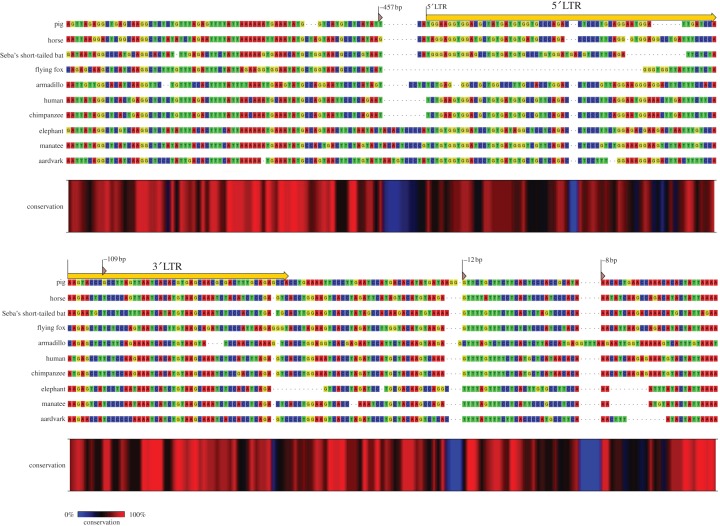
ERV-L integration site: (*a*) 5′ integration site, showing the extreme end of the 5′ LTR of the ERV-L insertion in 10 mammalian species, together with flanking sequence. (*b*) Corresponding 30 integration site, showing the extreme end of the 3′ LTR, together with flanking sequence. A conservation colour gradient beneath the nucleotides indicates the level of sequence homology across all 10 mammals, with red showing highest conservation. Triangular flags indicate segments of DNA deleted from a particular species for illustration purposes. Because alignment was poor at these regions, the golden mole was omitted (full flanking region alignments are given in the electronic supplementary material).

When aligned, the 5′ and 3′ flanking regions generally showed considerable levels of homology across all species (figure 1). However, approximately 450 bp of sequence just upstream of the 5′ LTR was found to contain a large insertion in the Afrotheria (which was subsequently excised from figure 1). Upstream of this 450 bp region, sequence conservation was high across all species (see additional flanking sequence alignment in the electronic supplementary material). Figure 1 indicates that the ERV-L sequence from each species is therefore likely to have integrated at the same locus in the genomes of multiple placental mammals (the golden mole sequence is, however, missing from the 5′ flank alignment, owing to the absence of this region from the genome database). Because retroviral integration is generally random [30], this provides strong evidence for the orthology of the element.

### (b) Full-length viral sequence alignment and identification of shared selfish genetic elements

**Figure 2.**
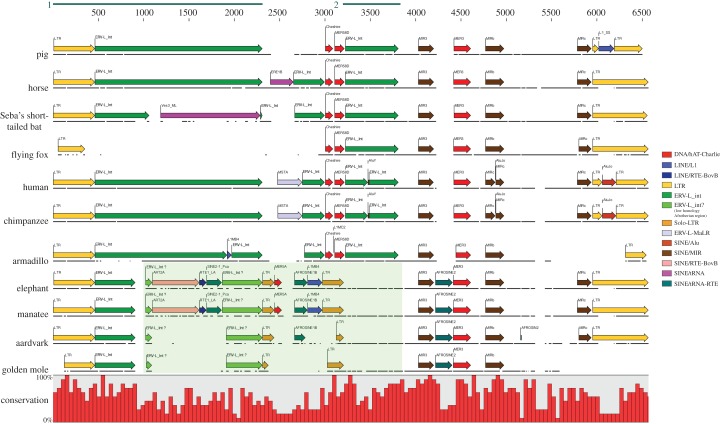
Schematic of ERV-L element and identified repeats: SGEs, identified by RepeatMasker, are individually flagged, with the repeat families being colour-coded. A solo-LTR, present only in the Afrotheria, is highlighted in beige, whereas a region of low homology in the same mammalian superorder is shaded light green. The presence of nucleotide sequence is indicated by horizontal black lines, to make obvious where gaps have been inserted in order to maintain the alignment. Sequence conservation is indicated by a histogram running underneath the alignment. Region 1 and region 2 are demarcated by dark lines running above the alignment, and correspond to the regions used for phylogenetic analysis (region 1 corresponds to figure 5; region 2 corresponds to a second cophylogeny given in the electronic supplementary material).

**Figure 3.**
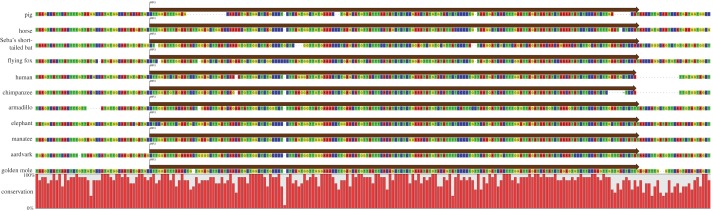
Orthologous MIR3 retrotransposon: an MIR3 retrotransposon, and flanking DNA, shared across all 11 mammalian species. The nucleotide sequence beneath each annotation is shown, with a graph running beneath the alignment to indicate site-specific sequence conservation across the taxa.

**Figure 4.**
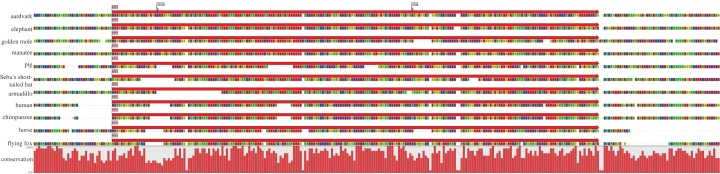
Orthologous MER3 DNA transposon: a non-autonomous MER3 hAT-Charlie DNA transposon, and flanking DNA, shared across all 11 mammals. The nucleotide sequence beneath each annotation is shown, with a graph to indicate site-specific sequence conservation across the taxa. Flags indicate deletions that were made for illustrative purposes, in regions that contained unique insertions.

Prior to alignment, the element was verified as an ERV-L element, although phylogenetic analyses demonstrated the element to be basal to other HERV-L elements selected from the human genome (A. Lee & M. Tristem 2013, unpublished data). The ERV elements were found to be highly mutated, because they did not encode any intact retroviral ORFs. To further demonstrate orthology, the proviral DNA of each species was aligned and annotated with mammalian repeats identified by RepeatMasker [25]. This revealed that many SGEs were shared across all the investigated species, at similar positions. Figure 2 shows high conservation at both 5′ and 3′ LTRs across all species and the internal region was shown to be well conserved at several points, most notably between approximately 500 and 1000 bp (excepting the Armadillo, in which there have been deletions), and between approximately 3200 and 3800 in the Boreoeutherian mammals. Two separate MIR elements, at positions approximately 4000 (figure 3) and approximately 4750, and an MER3 at approximately 4450 (figure 4) are shared across all 11 mammals. Additionally, another MIR element (at position approx. 5800) was shared across nine mammals; the corresponding sequence is missing from the armadillo and golden mole for this MIR (probably as the result of subsequent deletion of genomic DNA in these host species).

The green shaded area in figure 2 demarcates a region in which the Afrotheria shared very little sequence homology with the Boreoeutherian mammals. This region represents approximately 2.5 kb of the alignment. An additional LTR structure was also identified within this region, at positions approximately 2300–3200 bp in the Afrotheria. This LTR was itself interrupted by the insertion of a MER5A and L1MB4 repeat in the sirenians, and an AFROSINE1B in both sirenians and the aardvark. Phylogenetic analysis confirmed this to be a solo-LTR structure (A. Lee & M. Tristem 2013, unpublished data). This was most likely the result of a secondary viral integration within the existing ERV-L element, and its subsequent recombinational deletion, leaving behind a single 5′ solo-LTR structure [1].

In addition to the region of poor similarity in the Afrotheria, there was an approximately 800 bp region where RepeatMasker failed to identify any repeats (or similarity to other ERV-L sequences); this is present in all 11 mammalian sequences, starting approximately 5000 bp into the alignment (figure 2). As all the sequences exhibit significant homology to each other throughout this region, it is unclear whether this is a heavily mutated internal region, or whether it is an unexplained insertion. Regardless, this change in the ERV-L genome is expected to have occurred prior to the Afrotherian–Boreoeutherian divergence, because it is evident in all 11 placental mammals. This region is flanked on either side by two conserved MIR repeats.

### (c) Sequence identities

We calculated the percentage shared sequence identities between ERV-L orthologues in pairs of taxa across two regions, from positions 1–997 (region A) to 4417–5247 (region B). Table 1 indicates relatively good sequence conservation between the most distant two superorders, I and IV, with up to 65% identities shared between species such as Seba's short-tailed bat against the manatee, in region A. Similarities for region B were generally equivalent. Sequence identities for some taxa, especially in region A, however, are far lower for the golden mole, ranging from 25 to 50%, relative to superorders II-IV; this would indicate a more divergent 5′ LTR in these taxa. Similarly, pairs of armadillo against superorders III and IV, show a lower range of shared identities, between 34 and 39%.

**Table 1.** Pairwise observed sequence identities (%) for two regions: region A is shaded blue, and region B is red. Region A covers the first 997 bp of the element, including the 5′ LTR and ERV internal region. Because the flying fox was missing sequence data in this region, it has been omitted. Region B covers a 830 bp segment of the alignment, starting at position 4417. The full alignment is available in the electronic supplementary material. Blue, region A; red, region B; grey, omitted data.

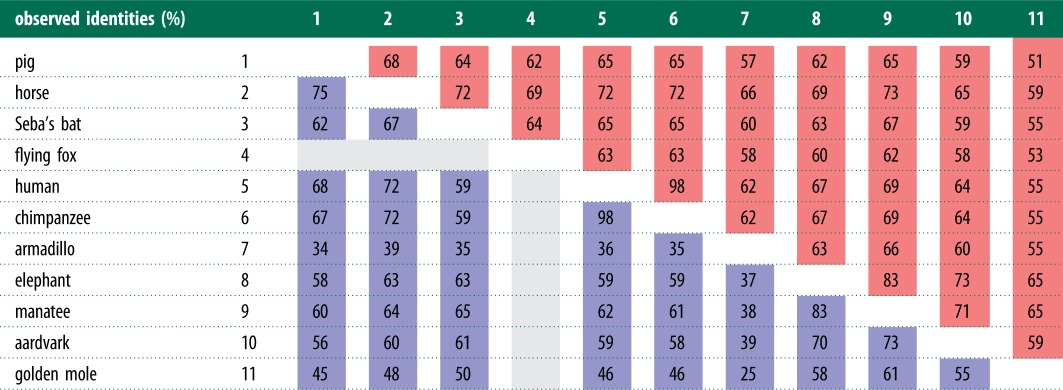

### (d) Phylogenetic analysis

**Figure 5.**
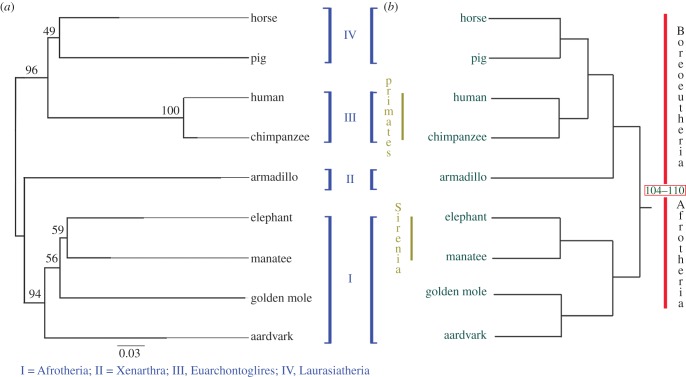
Retroviral-mammalian cophylogeny: cophylogeny comprising an unrooted maximum-likelihood phylogenetic tree constructed from the described ERV-L element (*a*) and an adapted schematic rooted mammalian phylogeny (*b*) [31–34]. Species falling into each of the four mammalian superorders are bracketed in blue, while the Afrotherian and Boreoeutherian clades are indicated in red; primates and Sirenia are indicated in beige. Branch lengths on the retroviral tree (*a*) are scaled to indicate the number of substitutions per site, and bootstrap values are shown. The initial divergence of the placental mammals into Afrotheria and Boreoeutheria is indicated in a box [31,36].

We constructed a cophylogeny of the ERV-L sequences together with a mammalian phylogeny adapted from the literature (figure 5). This demonstrated that the retroviral phylogenetic relationships resolve well to those shown in the existing mammalian phylogeny [37], as would be expected with orthologous elements. There are, however, topological differences between the Xenarthran and Afrotherian mammals. This is not surprising given the previous difficulties of reconstructing mammalian phylogenies from single genes [38].

### (e) Estimating the initial integration date

The orthologous nature of the ERV-L sequences indicates that the virus first integrated into an ancestor of all extant placental mammals, which started diverging between 104 and 110 Ma [31,36]. Thus, the virus has the same minimum age. However, it could also be considerably older, as is suggested by multiple shared SGEs, which presumably integrated into the ERV-L element itself prior to the divergence of the placental mammals. To investigate this, we examined the similarity between various combinations of the ERV-L 5′ and 3′ LTRs.

Upon integration, both viral LTRs generally comprise identical sequences. Thus, a comparison of the percentage identity between any 5′ LTRs (or any 3′ LTRs) indicates how much LTR sequence divergence has occurred since the earliest divergence of the placental mammals. By contrast, a calculation of the percentage divergence between a 5′ LTR and a 3′ LTR indicates how much sequence divergence has occurred since the initial integration event. Using Needle, a pairwise comparison was made between the elephant and human 5′ LTR sequences, and a divergence of 31% was given between them. We then compared the 5′ LTR of the elephant ERV-L with the 3′ LTR of the human element (and vice versa), and obtained an average divergence of 53%. Thus, 53 − 31% = 22%, meaning that 22% of the sequence divergence between the 5′ and 3′ LTRs likely occurred prior to divergence of the placental mammals, 104–110 Ma [31,36,39].

It is problematic to turn percentage 5′ versus 3′ LTR divergence data into timescales, owing to the lack of data regarding the neutral mutation rates of ancient mammals. Gene conversion and recombination between LTRs can also make these estimates unreliable. However, if we assume the ancestral placental mammal was small, with a relatively fast generation time, and therefore apply a conservative neutral mutation rate based on the mouse genome, of 4.5 × 10^–9^ substitutions per site per year [40], we find an initial integration date of approximately 128 Ma (22 divided by (0.45 × 2) + 104).

These calculations were then repeated for human against armadillo ERV-L LTRs, and human against horse ERV-L LTRs, giving initial integration date estimates of approximately 140 and approximately 139 Ma, respectively. We therefore suggest that the initial integration date could have preceded the divergence of the placental mammals by at least 24 Myr, and perhaps by up to 36 Myr.

### (f) *d*_N_/*d*_S_ ratios

HyPhy was used to calculate individual values for *d*_N_ and *d*_S_, per codon, across the seven Boreoeutherian mammals. We were unable to reject the null hypothesis of neutrality (*p* = 0.71), as would be expected in the case of uncoopted, orthologous insertions, but which is often not the case for paralogous insertions [41].

### (g) Synteny

**Figure 6.**
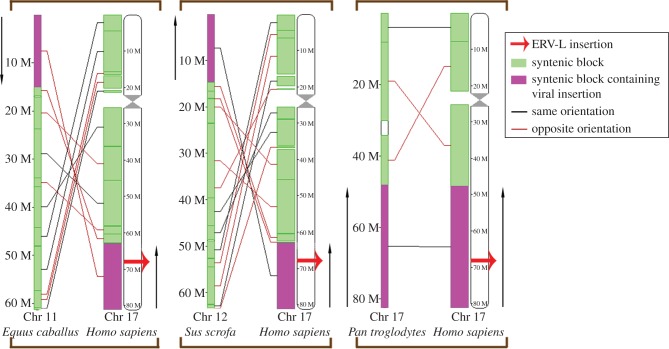
Syntenic relationship between human chromosome 17 and three selected mammals: a chromosome synteny map adapted from ENSEMBL's SyntenyView, showing syntenic blocks (shaded green) shared between human chromosome 17, where the ERV-L insertion occurs (highlighted in red), and corresponding chromosomes in the horse, pig and chimpanzee. Syntenic blocks containing the ERV-L insertion are shaded pink.

The human ERV-L insertion is present on chromosome 17. Analysis of synteny across this entire chromosome was performed relative to the horse, pig and chimpanzee. While the order is scrambled, as would be expected between distantly related species, figure 6 shows a high number of syntenic blocks shared between chromosome 17 of the human genome and corresponding chromosomes in each of the remaining three species. Furthermore, the syntenic block containing the virus was shown to be present in the same orientation in all species, except for the horse where it is inverted. This analysis is not yet available for any of the Xenarthran or Afrotherian genomes.

## 4. Discussion

### (a) Orthology of the endogenous retrovirus sequences

This is the first time an ERV, or any other type of virus, has been demonstrated to exhibit orthology across all four Eutherian superorders, making this the oldest individual viral sequence examined to date. Within the Afrotheria, the ERV-L element was recovered from the genomes of the African elephant, manatee, aardvark and golden mole. Furthermore, the presence of additional orthologues in multiple Boreoeutherian hosts indicates that the initial infection event predates the earliest divergence of the placental mammals, estimated to have occurred approximately 104–110 Ma [31,36]. Prior to this study, ERV-L elements have previously been found across the placental mammals at low copy numbers, strongly implying a minimum age of 70 Myr for this retroviral family [6]. These findings, however, did not attempt to identify any orthologous retroviral elements. While several other studies have examined orthologous retroviral families between primate species [42], none have extensively described retroviral insertions covering the breadth of mammalian species studied here.

In this investigation, multiple lines of evidence pointing to the orthologous nature of the ERV-L element were found. Because retroviruses generally insert at random within host genomes, only orthologues, but not paralogues, would be expected to share similar host flanking sites. The investigated ERV-L sequences from the different hosts all contained flanking sites with strong sequence similarity and, where synteny data were available, were found to have inserted into the same region of their respective genomes.

We estimated that the initial integration event occurred tens of millions of years before the first divergence of the placental mammals studied. Consistent with this, we found that several SGEs are present in the same locations in all the ERV-L sequences. These would therefore have first inserted within an ancestral mammal after the initial integraton event of the ERV-L, but before the placental mammals started radiating. In particular, three SGEs were identified by RepeatMasker [25] to be conserved across most or all of the host taxa. In each case, the SGEs belong to the same family, and are in the same orientation with respect to the ERV-L. Furthermore, sequence alignments demonstrated that the same region of each SGE is present at almost exactly the same location within each of the ERV-L sequences.

These additional SGEs are therefore also some of the oldest discovered to date. Previous to this study, MIR elements were estimated to be 130 Myr old. However, orthology was only demonstrated to 65 Ma, with the remaining age estimated through the aid of a molecular clock [18]. Other studies that have previously investigated similar orthologues identified a *Can* SINE element with a minimum age of 40 Myr [43]. Moreover, SINE elements have also been used to reconstruct ancient orthologies; such an example is the use of *Hpa*I SINEs as clade markers for the study of salmonoid fish evolution [44,45]. Other studies have recovered the complete chronology of Alu elements in primates, using specifically developed computational algorithms [46].

Once a retrovirus inserts into a host genome it remains at that location. For this reason, selection often operates differently on orthologous versus paralogous ERVs. When a retrovirus integrates into the germline and is inherited by host progeny, it is no longer under selection, because that copy remains at the same genomic locus, and the provirus mutates as a result of the host's DNA replication error rate (unless it becomes co-opted). However, when virus particles are produced (either extra- or intracellularly), the replicating retrovirus pool remains under negative selection until one of the viruses successfully inserts into, and becomes fixed within, the germline again. Thus, purifying selection operates on the viral genome between germline integration events [41]. There may be many hundreds of replication cycles within somatic cells between each germline integration event [8]. Therefore, considering the ERV-L element being studied as orthologous, a *d*_N_/*d*_S_ ratio of unity would be expected. A greater number of synonymous substitutions over non-synonymous substitutions indicates negative selection, resulting in a ratio of less than unity. We analysed the *d*_N_/*d*_S_ ratios between the ERV-Ls in this study and were unable to reject the null hypothesis of neutrality, further supporting the orthologous nature of the elements. This also suggests that there has been no recombination with different ERV-L sequences (while maintaining the same flanking sequences) within a particular host genome, which would also be expected to reduce *d*_N_/*d*_S_ ratios.

Individual ERV-L elements do not change location within the host genome, and evolve neutrally. They would therefore be expected to track a phylogeny of their host organisms. Thus, phylogenies of the virus and its hosts should be congruent with one another. We constructed phylogenies of parts of the ERV-L sequences and found that they did indeed almost match the host phylogeny. Taken together, all these lines of evidence clearly indicate that the ERV-L sequences investigated here are orthologous in nature.

### (b) Sequence conservation over time

As expected, observed sequence identities shared between species served to highlight the importance of selecting taxa with slower generation times when reconstructing ancient orthologies. For instance, it was observed that the range of percentage identities for pairwise comparisons containing smaller mammals was generally lower than with larger mammals. Such examples included pairs containing armadillo and golden mole, when compared against superorders III and IV. These differences are most likely the result of a higher neutral evolution rate in these smaller mammals. Conversely, the larger mammals with slower generation times, such as the elephant and manatee, contained ERV-L sequences accruing mutations at a comparatively slower rate. The viral insertions in these mammals have a higher range of shared identities when compared with other mammals in superorders II–IV. On some occasions ERVs, or parts of ERVs, can persist for long periods because they have been co-opted [47,48]. We can rule out this possibility here because of the multiply defective nature of the ERVs; all of them contained numerous in-frame stop codons and frameshifting indels. Co-opted elements are usually under selection to maintain an intact ORF.

### (c) Difficulties encountered when reconstructing ancient orthologies

Global genomic databases are constantly growing, amassing into a widely varied library of genomic sequences. One of the many obstacles encountered in reconstructing ancient orthologies is the nature of the required sequence information. First, the full-length viral insertion must be retrieved together with sufficient 5′ and 3′ flanking sequence for demonstrating the orthology of the element. This can often mean that a single, intact DNA sequence upwards of 10 kb is required. While some genomes have been sequenced to completion, many others comprise unassembled reads and, depending on the sequencing technology used, individual contigs and reads may not be long enough to prove that a virus is orthologous. This limitation resulted in the elimination of certain species considered for this study. It is also important to study genomes from taxa that have relatively low neutral mutation rates to ensure a higher likelihood of orthologue conservation across multiple taxa.

Other problems revolve around the specificity and stringency of the alignment algorithms used to retrieve corresponding viral elements from genomic sequence libraries. NCBI's BLAST search, for instance, while accurate, can often be too stringent to uncover matches in highly divergent sequences. This can potentially lead to viral orthologues remaining undetected. A number of alternative alignment algorithms are available, but many of the most sensitive programs, in terms of detecting weak sequence similarity, cannot compute sufficient amounts of data in the same way that BLAST can search an entire genomic record in a relatively short space of time. Another of BLAST's limitations is its low sensitivity to the presence of large insertions or deletions. In such cases, alternative algorithms such as BlastAlign [49] are superior in retrieving homologous sequences. Thus, during this investigation, combinations of algorithms were used: BLAST, with its ability to perform fast, genome-wide screens, together with MUSCLE and Needle, with their heightened sensitivity and accuracy [24,27]. We also found that RepeatMasker was generally very efficient at identifying ERV and other SGE sequences.

This study has presented a pan-Eutherian viral orthologue, demonstrating the oldest single endogenized virus identified to date. It has also been possible to identify some of the oldest described individual SGEs, setting a new benchmark for studying orthologous elements within the field of molecular paleovirology. While it is possible that older orthologues may be identified, the problems discussed above suggest that we may be approaching the maximum achievable lookback time—at least as far as the mammals are concerned. It may be possible, although very difficult, to identify orthologous insertions between placental mammals and marsupials (both owing to the divergence date of 147 Ma [37], and also to relatively fast rates of neutral evolution in the extant marsupials) but the placental and monotreme divergence date (167 Ma [37]) may remain beyond reach.
